# Characterization of the ferret TRB locus guided by V, D, J, and C gene expression analysis

**DOI:** 10.1007/s00251-019-01142-9

**Published:** 2019-12-03

**Authors:** Bram Gerritsen, Aridaman Pandit, Fatiha Zaaraoui-Boutahar, Mirjam C. G. N. van den Hout, Wilfred F. J. van IJcken, Rob J. de Boer, Arno C. Andeweg

**Affiliations:** 1 Theoretical Biology and Bioinformatics, Utrecht University, Utrecht, the Netherlands; 2 Department of Pathology, Yale School of Medicine, New Haven, USA; 3 Rheumatology and Clinical Immunology, University Medical Center Utrecht, Utrecht, The Netherlands; 4 Department of Viroscience, Erasmus Medical Center, Rotterdam, The Netherlands; 5 Center for Biomics, Erasmus Medical Center, Rotterdam, The Netherlands

**Keywords:** T cell receptor, TRB locus, Ferret (*Mustela putorius furo*), IMGT, Comparative genomics, Immune repertoire sequencing

## Abstract

The domestic ferret, *Mustela putorius furo*, is an important mammalian animal model to study human respiratory infection. However, insufficient genomic annotation hampers detailed studies of ferret T cell responses. In this study, we analyzed the published T cell receptor beta (TRB) locus and performed high-throughput sequencing (HTS) of peripheral blood of four healthy adult ferrets to identify expressed V, D, J, and C genes. The HTS data is used as a guide to manually curate the expressed V, D, J, and C genes. The ferret locus appears to be most similar to that of the dog. Like other mammalian TRB loci, the ferret TRB locus contains a library of variable genes located upstream of two D-J-C gene clusters, followed by a (in the ferret non-functional) V gene with an inverted transcriptional orientation. All TRB genes (expressed or not) reported here have been approved by the IMGT/WHO-IUIS nomenclature committee.

**Electronic supplementary material:**

The online version of this article (10.1007/s00251-019-01142-9) contains supplementary material, which is available to authorized users.

## Introduction

The ferret (*Mustela putorius furo*) is an important mammalian model species to study human respiratory infections. Ferret infection models are well suited to study the pathogenicity and transmissibility of Coronaviruses (SARS), Pneumoviridae (RSV) and Orthomyxoviruses that include human and avian influenza viruses (Enkirch and von Messling 2015; Oh and Hurt 2016). Ferrets are an attractive mammalian model species for these infections since ferrets and humans share similar lung physiology, and most notably a very similar (viral) receptor distribution throughout the respiratory tract (Belser et al. 2011; van Riel et al. 2006). A significant drawback of the ferret model is a lack of ferret specific reagents for detailed studies of the host immune response to these pathogens. Nevertheless, the use of the ferret model has increased over the years, and its usage, with the recent publication of the ferret (draft) genome (Peng et al. 2014), is likely to increase even further. Currently, little is known about the T cell receptor (TCR) repertoire of the ferret, limiting the options to monitor the immune response of ferrets to experimental infections with influenza virus and other pathogens.

TCRs mediate recognition of peptide antigens presented to T lymphocytes via the peptide-MHC complex (Davis and Bjorkman 1988). Conventional TCRs are *αβ* or *γδ* heterodimers that are formed by somatic rearrangement of Variable (V), Diversity (D), and Joining (J) gene segments for the *β* and *δ* chains, and V and J gene segments for the *α* and *γ* chains (Davis and Bjorkman 1988). Although the ratio between *αβ* and *γδ* T cell subsets is not known for the ferret, the *αβ* T cells are much more common than *γδ* T cells in both human and dog (Mineccia et al. 2012). The *β* chain (at least in humans) tends to interact more closely with the peptide antigen than the *α* chain (Glanville et al. 2017), making the TRB locus the most interesting first candidate to annotate in detail.

In this study, we annotate the expressed V, D, J, and C genes in the ferret TRB locus by combining genomic information from the locus with HTS of the ferret TRB repertoire. We find that the TRB locus of the ferret has a similar structure to that of other mammalian TRB loci, such as mouse and human (Glusman et al. 2001), bovine (Connelley et al. 2009), dog (Mineccia et al. 2012), and rabbit (Antonacci et al. 2014): a library of V genes, followed by two (or three in bovine) D-J-C clusters. Each cluster consists of one D gene, six or seven (six in ferret) J genes, and a single C gene. The D-J-C clusters are followed by a V gene with an inverted transcriptional orientiation. We also performed a phylogenetic analysis, showing that the ferret V and J genes are indeed most closely related to those of the dog. The ferret locus is small like that of the dog, about 300 Kb, and has a (largely) conserved synteny with the dog TRB locus. Our annotation of the ferret TRB locus will enable detailed studies of T cell responses to support research on novel or improved antiviral strategies for influenza and other viral infections employing the ferret as a model organism. All TRB genes identified in our analysis (expressed or not) have been approved by the IMGT/WHO-IUIS nomenclature committee.

## Materials and methods

### Genome sequences

The ferret genomic scaffolds (GL896904.1 and GL897291.1) representing the TRB locus were retrieved from Genbank (ferret whole genome shotgun sequence Mus-PutFur 1.0, ref 5) guided by sequence homology with the dog TRB locus (chro- mosome:CanFam3.1:16:6706526:7027700:1). The blast algorithm (Altschul et al. 1990) and Mauve (Darling et al. 2004) software were applied to align the ferret genome scaffolds with the dog genome sequence.

### Animals

Four surplus cryopreserved healthy control blood samples were obtained from an influenza vaccination-challenge study (Bodewes et al. 2010). The control blood samples originated from four 6 to 12 months old healthy outbred female ferrets.

### RNA isolation and 5′-RACE

Peripheral blood mononuclear cells (PBMCs) were isolated from ferret blood using a standard Ficoll gradient separation protocol. Subsequently, total RNA was isolated and purified using the RNeasy Mini Kit (Qiagen, Hilden, Germany): 250 μl of ethanol was added to the upper aqueous phase of the processed TRIzol samples and directly transferred to the RNeasy spin columns for purification. RNA concentrations and OD 260:280 nm ratios were measured with the NanoDrop^R^ ND-1000 UV-VIS spectrophotometer (NanoDrop Technologies, Wilmington, USA). TCR amplification was performed according to a protocol described by Mamedov et al. (Mamedov et al. 2013). Primer sequences are provided in Table 1. Various nested primer 2 primers were used since local sequence conservation is not yet established. Briefly, RNA obtained from unsorted PBMCs was reverse transcribed by RACE using a primer directed to the constant region. Twelve nucleotide long unique molecular identifiers (UMIs) were incorporated during cDNA synthesis (Kivioja et al. 2011). Subsequently, two-stage seminested and barcoded PCR amplification was performed including a size selection/agarose gel purification step after the first PCR (Mamedov et al. 2013).

**Table 1:** Primer sequences

Template switch primer	AAGCAGTGGTATCAACGCAGAGNNNNNNNNNNNNCTTGGGGG
FTRB-31	CAGTAGCTGGAGTCATTCT
Step-out primer 1	CACTCTATCCGACAAGCAGTGGTATCAACGCAG
FTRB-1	CACGTGGTCGGGGWAAAAGC
Step-out primer 2	NNNNCACTCTATCCGACAAGCAGT
FTRB-9F	TCTCTGCTTCTGACGGTTCAAAC
FTRB-9F1	GAGGTTTGACCTTTTCCAGATTCTC
FTRB-9F2	GGAGGTTTGACCTTTTCCAGATTC
FTRB-9F3	CTGTGACCGTGGGAGGTTTG
FTRB-9F4	TGTGACCGTGGGAGGTTTGAC

### TRB transcript sequence analysis

The resulting TCR amplicons were subjected to high-throughput sequencing ac- cording to the instructions of the manufacturers using the Ovation Low Complexity Sequencing System kit from NuGEN (San Carlos, CA, USA) and the Illumina MiSeq or the Hiseq2500 platforms both using indexed paired end 300 cycle runs. All sequence reads having the same UMI were collapsed into consensus sequences using the RTCR pipeline (Gerritsen et al. 2016). The BLAST+ (Camacho et al. 2009) and exonerate (Slater and Birney 2005) (version 2.2.0) software were used to align the consensus sequences with the ferret genomic scaffolds to identify V, D, and J genes. After describing the V and J sequences in the ferret TRB locus, the RTCR pipeline was used to annotate the sequences, and perform additional error correction.

## Results

### Identification of expressed ferret TRB V, D, J, and C genes

**Fig. 1:**
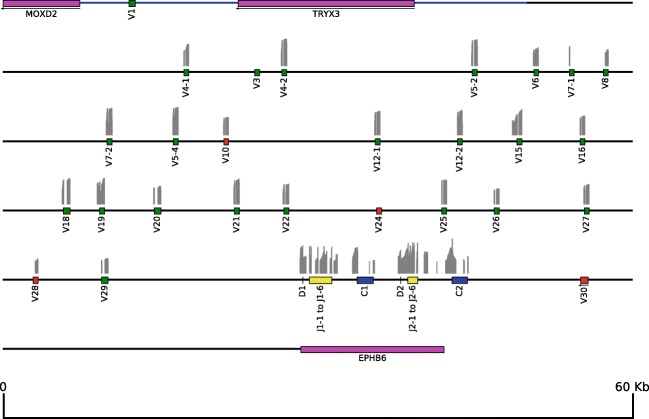
Schematic representation of the genomic organization of V, D, J, and C genes in the ferret TRB locus. The TRB locus is covered by scaffolds GL896904.1 and GL897291.1, here shown as blue and black horizontal lines, respectively. Boxes are to scale and show the following regions for the various genes: V genes (functional or ORF, green; pseudogenes, red), from the 5′ start of L-PART1 to the 3′ end of V-REGION; for D genes (black), the D-region; for J genes (yellow), from 5′ start of the J-REGION of TRBJ1-1 until 3′ end of the J-REGION of TRBJ1-6 and similarly for TRBJ2; for C genes (blue), from 5′ start of EX1 until 3′ end of EX4. A few non-TRB genes are included: the canonical start and end genes of the TRB locus, MOXD2 and EPHB6, respectively, and TRYX3. Genomic coverage from the HTS (gray bars; bar width is 10 bp) is shown on a log10 scale, excluding bars that have less than 50*×* coverage on average.

Consensus sequences, formed by collapsing all sequence reads sharing the same UMI (here referred to as a “UMI-group”), were aligned against the ferret draft genome. Most consensus sequences (> 99% for primers 9F1-9F4, > 50% for primer 9F) of UMI-groups containing more than 1 sequence read targeted the region between the ferret MOXD2 (Ensembl ID ENSMPUG00000002940.1) and EPHB6 (ENSMPUG00000008478.1) genes. This result is in line with the genome location of the TRB regions of a diverse set of mammals, i.e., cattle, human, mouse, and dog, of which the TRB regions are also flanked by MOXD2 and EPHB6 (Antonacci et al. 2014). Consensus sequences from UMI-groups of size 2 and up were aligned against the ferret genome. Next, we searched in the TRB locus for expressed V, D, J, or C genes in the regions with over 50 coverage (see Fig. 1).

**Fig. 2:**
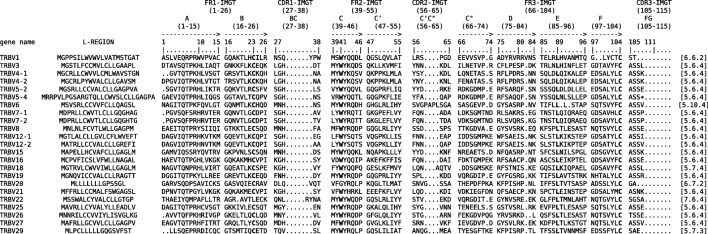
Protein display of the ferret consensus TRBV genes, showing only genes classified by IMGT as functional, F, or open reading frame, ORF. Alignment of the V-REGIONs, performed using DomainGapAlign (Ehrenmann et al. 2010; Ehrenmann and Lefranc 2011), is displayed according to IMGT unique numbering for V-REGION (Lefranc et al. 2003), and the amino acid length of CDR-IMGT is indicated in square brackets

Using the HTS data, we were able to identify 23 ferret TRBV genes that had over 50 x coverage over part of their region. Reads aligning to the same region were collapsed, and the resulting consensus sequence was used to identify the various features of a V-GENE, such as the location of the V-RS, SPLICE-DONOR, and SPLICE-ACCEPTOR sites. In cases where the consensus was unclear, either due to low coverage and/or poor agreement, we used the LIGMotif programme (Lane et al. 2010) in combination with manual curation to identify the various gene features (Fig. 2). In total, we identified 27 TRBV genes, of which IMGT classified 20 as functional (74%), 4 as pseudogenes (15%), and 3 as an ORF (see Figure S5 for genomic locations of V, D, J, and C genes).

Interestingly, in the functional V gene TRBV19, we discovered a non-functional splice variant (see Figure S1 for an example), leading to a frameshift and the loss of the FR1 and CDR1 regions of the V-EXON. This splice variant occurs on average in more than 25% of the sequence reads that align to TRBV19 in each of the four ferrets. To be able to detect this non-functional variant, sequence reads should extend nearly 200 bp into the V-EXON region. If reads do not extend this far into the V gene, this may lead to an overestimation of the expression level of V genes having such non-functional splice variants. To this end repertoire sequencing experiments should use reads that are long enough to detect the splice variants, or perform a bias correction based on dedicated experiments quantifying the proportion of non-functional splice variants in the ferret population.

**Fig. 3:**
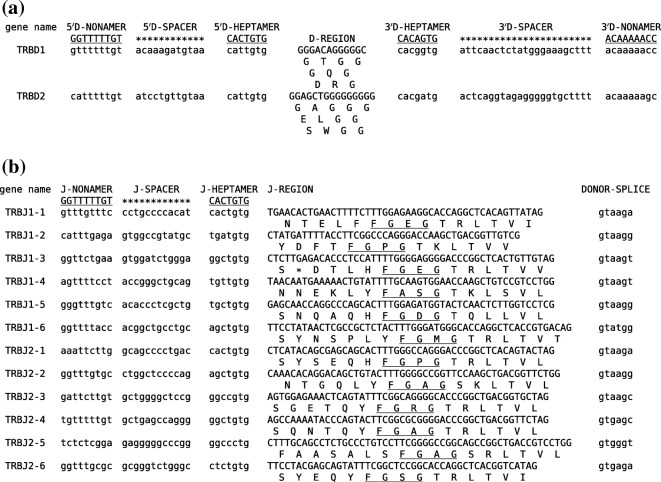
Nucleotide and deduced amino acid sequences of the ferret TRBD (**a**) and TRBJ (**b**) genes. TRBJ1-3 is classified as a pseudogene because of the presence of a stop codon (asterisk).

**Fig. 4:**
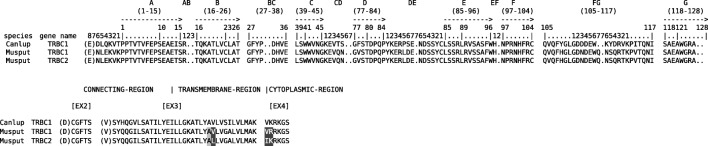
Protein display of the ferret TRBC genes, including dog TRBC1, according to the IMGT unique numbering for C-DOMAIN (Lefranc et al. 2005). Nucleotide differences between TRBC1 and TRBC2 exons of the ferret are shown in light gray and dark gray, for synonymous and non-synonymous substitutions, respectively

Similarly to the V genes, we manually curated the D, J, and C genes guided by the HTS data (Figs. 3 and 4). The general structural organization of the ferret TRB locus (see Fig. 1) is similar to that of the dog (see Ref. (Mineccia et al. 2012) for the locus of *Canis lupus familiaris*). Like in the dog, the ferret TRB locus spans about 300 Kb, which is much less than the 650 Kb long locus of humans. The ferret TRB locus contains a region of V genes followed by two D-J-C clusters. Both D-J-C clusters span about 7 Kb and consist of one D gene, six J genes, followed by a C gene. About 11 Kb downstream of the second D-J-C cluster, there is a TRBV gene (TRBV30) with an inverted transcriptional orientation. In the ferret, IMGT classified TRBV30 as a pseudogene because of a missing DONOR-SPLICE. Instead, the homologous TRBV30 in both human and dog is functional.

The ferret TRBD1 and TRBD2 genes are 12 bp and 15 bp long, respectively, and both genes are productively read in all 3 reading frames (Fig. 3a). The ferret J genes are between 44 and 53 bp long and conserve the FGXG motif, required for a functional J gene. The only exception is TRBJ1-4, classified as an ORF because it has a noncanonical J-MOTIF (“FASG”; Fig. 3b) identical to the homologous gene, TRBJ1-4, in the dog. Both TRBJ2-1 and TRBJ2-5 are also classified as an ORF due to having a noncanonical J-NONAMER. Although TRBJ1-3 is a pseudogene (because of a stop codon), it can still lead to functional transcripts by VDJ recombination, as it is used in about 3% of the TCRB clonotypes (Fig. 6b). Like other mammalian species such as human, mouse, dog, and rabbit (Antonacci et al. 2014), the ferret TRBC genes consist of 4 exons each (Fig. 4). The TRBC genes of the ferret are identical to each other for the first 2 exons (EX1 and EX2), and differ by only 2 nt in EX3 and also by 2 nt in EX4. The FG loop is one amino acid longer than the longest TRBC FG loop described by IMGT (Lefranc et al. 2005). We extended the numbering of the FG loop to accommodate the additional amino acid (Fig. 4). Both TRBC genes appear to be functional, having proper acceptor and donor splice sites for each exon, not containing any stop codon or frameshifts.

### Phylogenetic analysis of the ferret TRBV and TRBJ genes

**Fig. 5:**
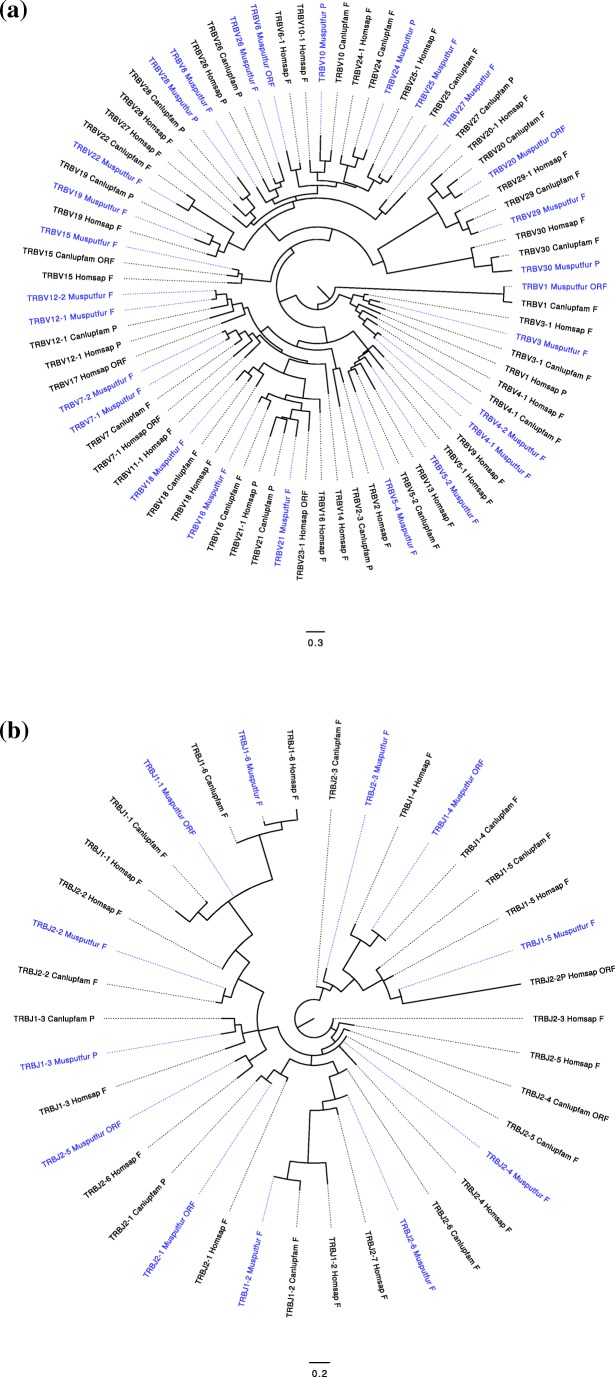
Phylogenetic trees of the dog, human, and ferret TRBV (**a**) and TRBJ (**b**) genes. Unrooted phylogenies were inferred from V-REGION (**a**) and J-REGION (**b**) amino acid sequences using the PhyML programme (Guindon and Gascuel 2003). The phylogenetic trees were visualized using FigTree (Rambaut 2009). Ferret genes are indicated in blue, human and dog genes in black

We aligned the ferret TRBV (V-REGION) and TRBJ (J-REGION) amino acid sequences to human and dog TRBV and TRBJ sequences, and constructed a phylogenetic tree using a maximum likelihood approach (Guindon and Gascuel 2003) (Fig. 5). We included both functional and non-functional ferret V and J genes. The ferret TRBV genes tend to cluster closer together with the dog than human TRBV genes. Most ferret TRBJ genes are more closely related to the dog TRBJ genes than to the human TRBJ genes, except for TRBJ1-5 and TRBJ2-6, which are closer to human TRBJ1-5 and TRBJ2-7, respectively.

### Analysis of the ferret TRBV and TRBJ usage

**Fig. 6:**
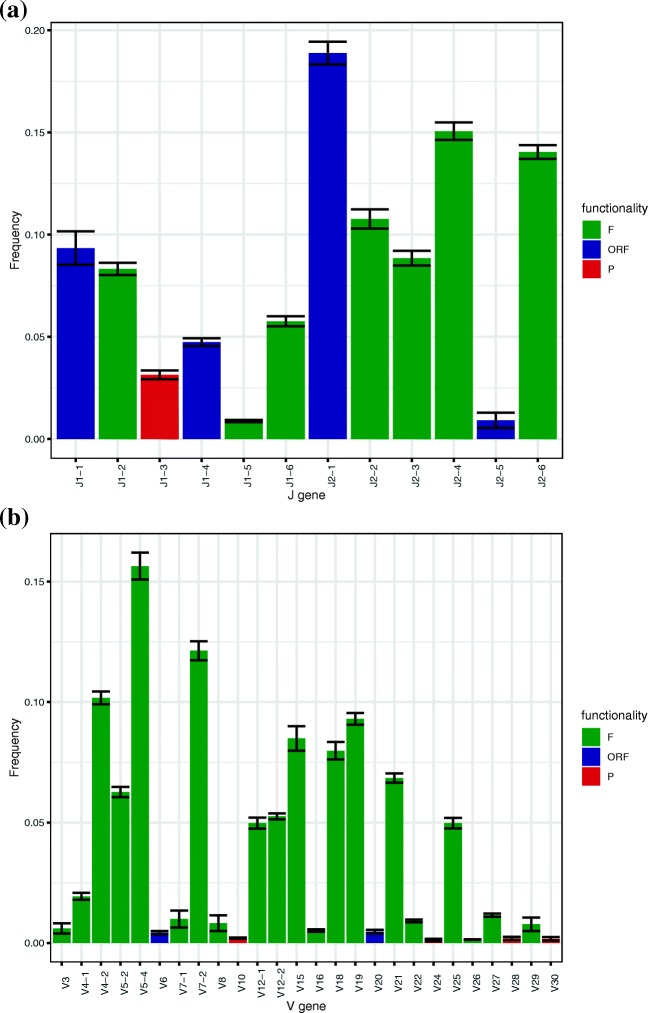
TRBV (**a**) and TRBJ (**b**) gene usage in the ferret. Bars indicate average fraction of distinct TCR sequences encoding a particular gene. Error bars indicate SEM across 16 HTS datasets (4 ferrets total, of which 3 ferrets are represented by 5 repertoire sequencing runs each)

After identifying the ferret TRBV and TRBJ genes, we used the RTCR pipeline to annotate and error correct the sequence reads. Overall, the TRBV and TRBJ usage is very similar among the four ferrets (Fig. 6). Interestingly, TRBV5-4, which contains a stop codon at the 3′ end of the CDR3, is the most common TRBV gene in the repertoire. Similarly, the pseudogene TRBJ1-3 also produces functional transcripts (i.e., about 3% of all transcripts; Fig. 6).

## Discussion

We combined HTS and genome analysis to describe the (expressed) T cell receptor genes in the TRB locus of the ferret. The genomic organization of the ferret TRB locus is very similar to that described in other mammals such as human, mouse, dog, and rabbit (Mineccia et al. 2012; Antonacci et al. 2014): the locus is flanked by MOXD2 and EPHB6 at the 5′ and 3′ ends, respectively, and consists of a library of V genes followed by two D-J-C clusters, followed by a V gene, which is non-functional in the ferret, with an inverted transcriptional orientation. Thus, the ferret confirms the strong organizational conservation of mammalian TRB loci. The ferret and dog TRB loci are closely related, because the ferret and the dog are in the same mammalian order, the Carnivora. Like in the dog, the ferret TRB locus is relatively small (300 Kb) and both contain about 20 functional TRBV genes. The ferret also expresses TRBV and TRBJ genes that contain stop codons, which nonetheless lead to functional transcripts, because these stop codons are deleted during VDJ recombination.

The ferret TRB locus is represented by two scaffolds of the draft genome assembly of the ferret as indicated in Fig. 1. Since, one, the highly conserved synteny with the dog TRB locus (any genomic sub-region of the dog TRB locus has a ferret counterpart on either of the two contigs), and two, the facing ends of the two scaffolds (Fig. 1, top line) display considerable sequence homology (> 1400 nt, data not shown), all ferret TRB VDJ genes are most likely contained in the current genome build. It is to be expected that the two scaffolds will be connected in a future build of the ferret genome when additional sequence information is available to complete genome regions encompassing gene families like the TRB locus that are particularly difficult to assemble correctly.

As previously described (Mineccia et al. 2012), the CDR3 length distribution is highly conserved, which in this study is also confirmed as the ferret and the human have nearly identical CDR3 length distributions (Supplemental Figure S2). Despite the relatively low number of V genes in the TRB locus of the ferret, the ferret repertoire is highly diverse as there is hardly any overlap in TCR*β* chains between the ferrets (Supplemental Figure S3 and S4). Within ferrets the TCR*β* chain overlap between samples is about 50%, probably reflecting the presence of memory clonotypes in the blood that have expanded due to antigen stimulation.

Although the ferret is an important animal model in research on respiratory infections, its adaptive immune responses were until now poorly characterized (Enkirch and von Messling 2015). Our characterization of expressed TRB genes in the ferret paves the way for detailed analysis of the cellular immune responses of ferrets in health and disease.

## Electronic supplementary material

Fig. S1 Protein sequence of a potential non-functional splice variant of TRBV19. The displayed sequence is from ferret GR5, and is a consensus of 774 sequence reads sharing the UMI shown on the left. “?” denotes an incomplete codon (here a single guanine), resulting in a frameshift.)

High resolution image (EPS 30 kb)

Fig. S2 Comparison of TRBV CDR3 length distribution of the ferret and the human. (red), average frequency of CDR3 lengths 1 through 30 across all ferret HTS datasets (11 TCR sequences longer than 30AA are not shown), (green), CDR3 length frequencies of a single human adult male (unpublished data; 174753 CDR3 lengths total, 46 TCR sequences longer than 30AA are not shown).

High resolution image (EPS 14 kb)

Fig. S3 Percentage overlap of CDR3 amino acid sequences between the different HTS datasets. The overlap between two samples was calculated using the Jaccard index, i.e. the fraction of total distinct CDR3 sequences that are shared between the two samples. The HTS datasets with the 9F primer (A) contained an order of magnitude more CDR3 sequences than the other datasets (B).

High resolution image (EPS 17 kb)

(B) (PNG 90 kb)

High resolution image (EPS 22 kb)

Fig. S4 Library statistics. (Size), number of reads containing a CDR3, and (Complexity), number of distinct CDR3 amino acid sequences (used in Figure S3 to calculate overlap between libraries).

High resolution image (EPS 40 kb)

Fig. S5 Description of the V (A), D and J (B), and C (C) genes in the ferret TRB locus. Genomic positions are relative to scaffold GL897291.1 or GL896904.1 (denoted with “*”).

High resolution image (EPS 58 kb)

(B)

High resolution image (EPS 47 kb)

(C)

High resolution image (EPS 44 kb)
