# Supplementary figures and images for: Characterization of the ferret TRB locus guided by V, D, J, and C gene expression analysis
Source: Immunogenetics. 2019 Dec 3;72(1):101–8. doi: 10.1007/s00251-019-01142-9 (PMC6971162; doi:10.1007/s00251-019-01142-9)

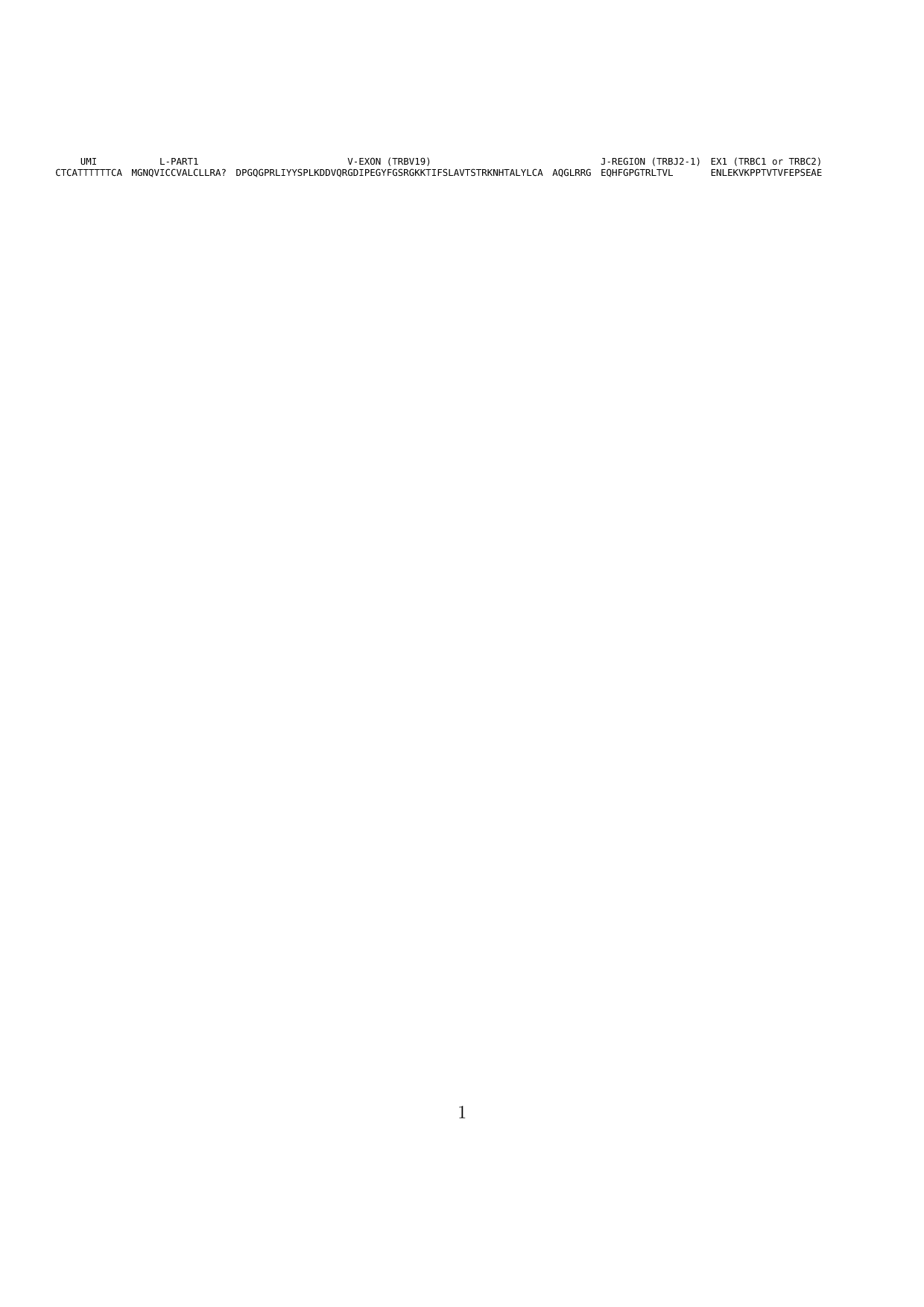

Supplement: Supplementary file 1 — Protein sequence of a potential non-functional splice variant of TRBV19. The displayed sequence is from ferret GR5, and is a consensus of 774 sequence reads sharing the UMI shown on the left. “?” denotes an incomplete codon (here a single guanine), resulting in a frameshift.) [file 251_2019_1142_Fig7_ESM.png]

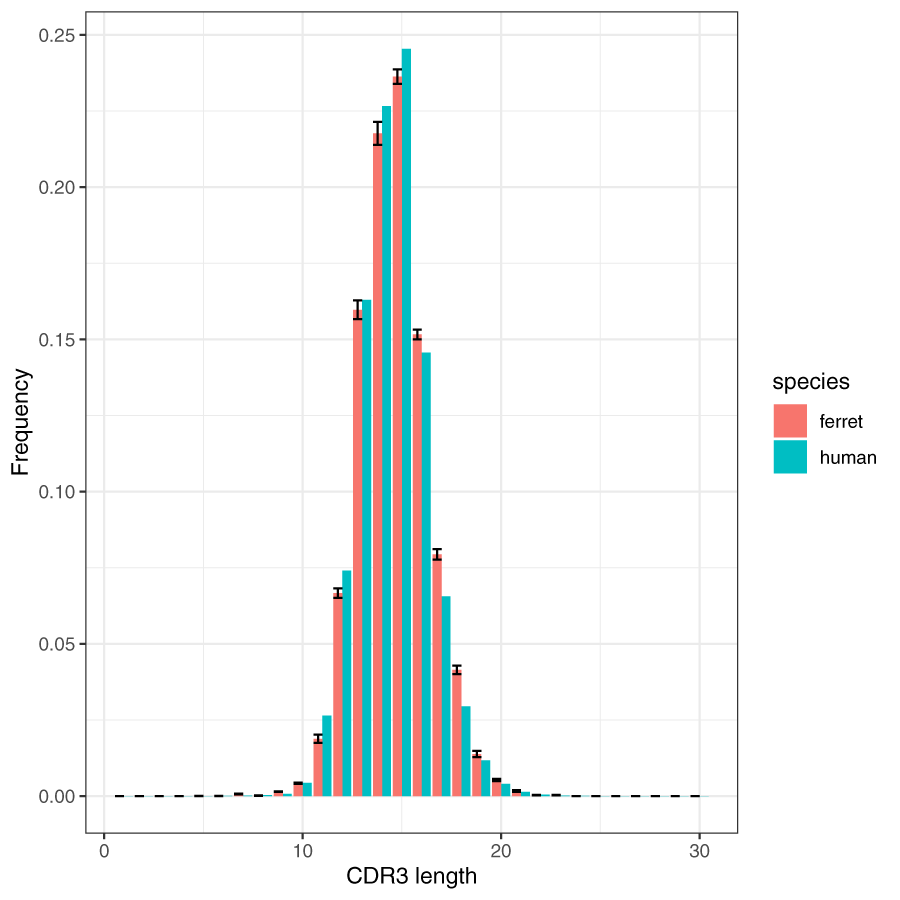

Supplement: Supplementary file 3 — Comparison of TRBV CDR3 length distribution of the ferret and the human. (red), average frequency of CDR3 lengths 1 through 30 across all ferret HTS datasets (11 TCR sequences longer than 30AA are not shown), (green), CDR3 length frequencies of a single human adult male (unpublished data; 174753 CDR3 lengths total, 46 TCR sequences longer than 30AA are not shown). [file 251_2019_1142_Fig8_ESM.png]

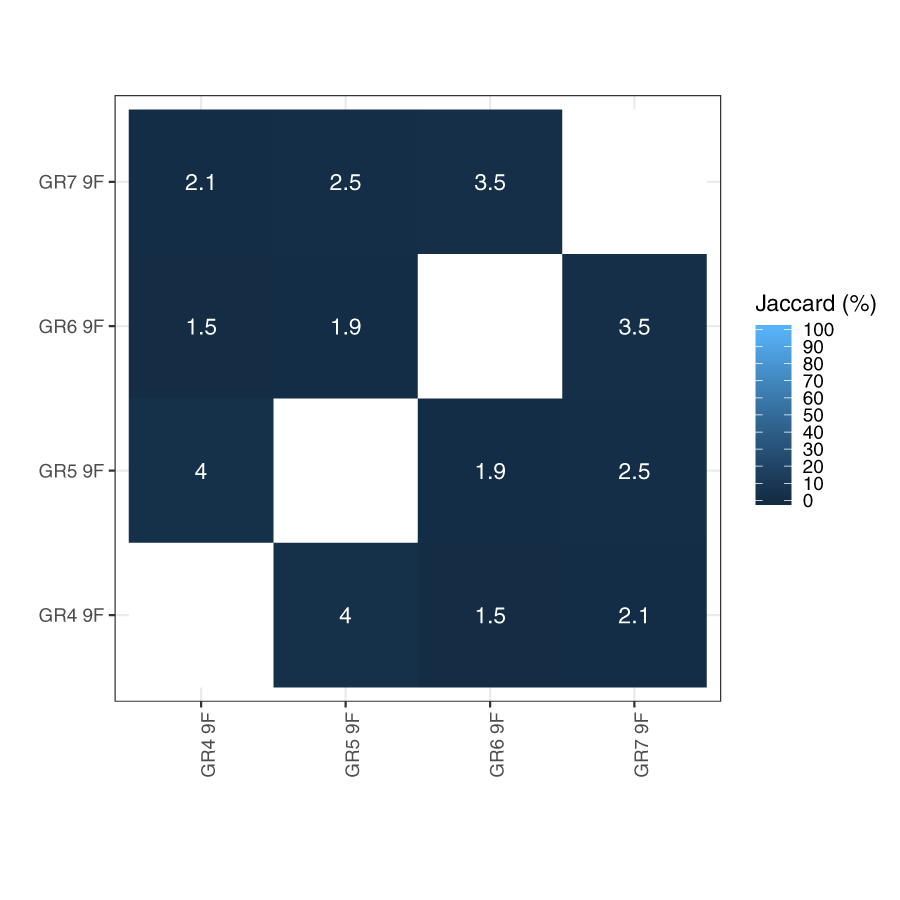

Supplement: Supplementary file 5 — Percentage overlap of CDR3 amino acid sequences between the different HTS datasets. The overlap between two samples was calculated using the Jaccard index, i.e. the fraction of total distinct CDR3 sequences that are shared between the two samples. The HTS datasets with the 9F primer (A) contained an order of magnitude more CDR3 sequences than the other datasets (B). [file 251_2019_1142_Fig9_ESM.png]

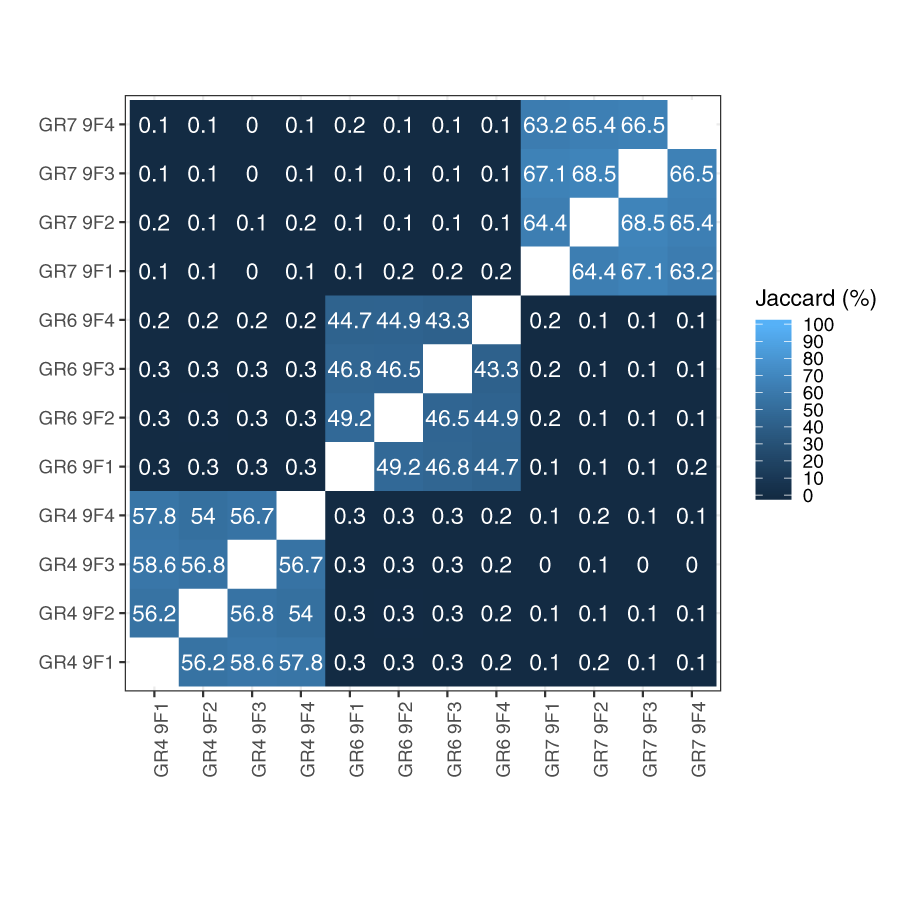

Supplement: Supplementary file 7 — (B) (PNG 90 kb) [file 251_2019_1142_Fig10_ESM.png]

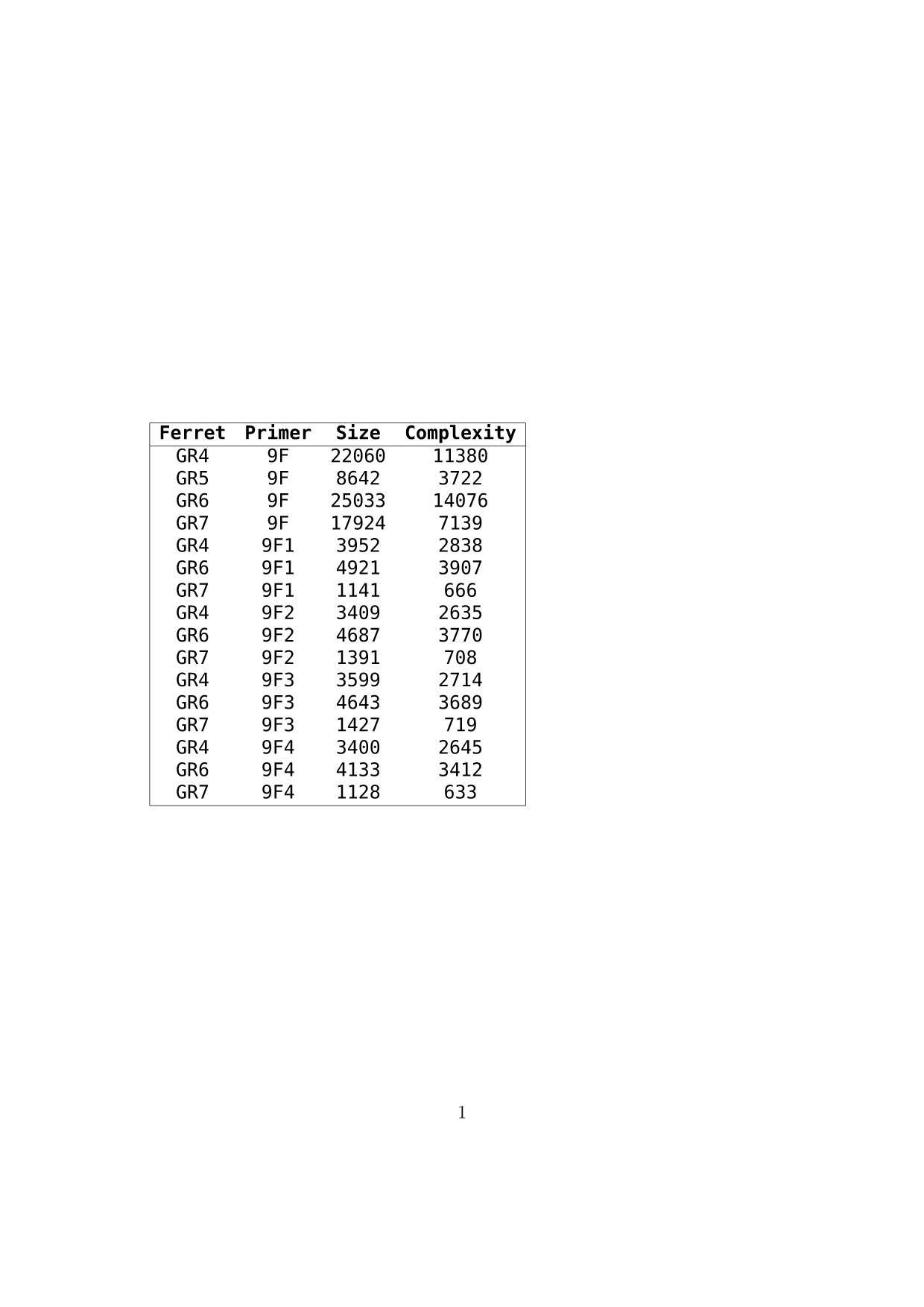

Supplement: Supplementary file 9 — Library statistics. (Size), number of reads containing a CDR3, and (Complexity), number of distinct CDR3 amino acid sequences (used in Figure S3 to calculate overlap between libraries). [file 251_2019_1142_Fig11_ESM.png]

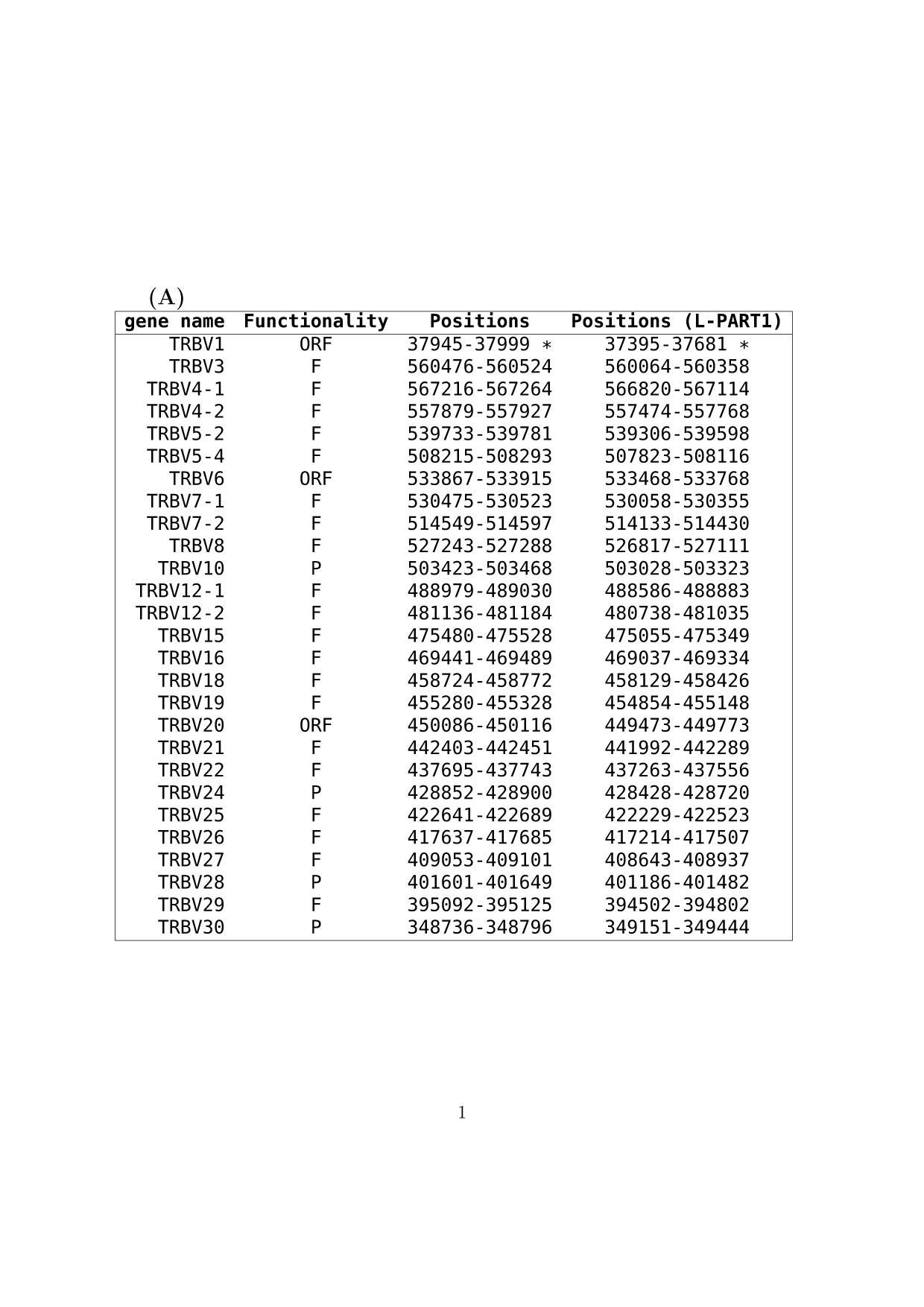

Supplement: Supplementary file 11 — Description of the V (A), D and J (B), and C (C) genes in the ferret TRB locus. Genomic positions are relative to scaffold GL897291.1 or GL896904.1 (denoted with “*”). [file 251_2019_1142_Fig12_ESM.png]

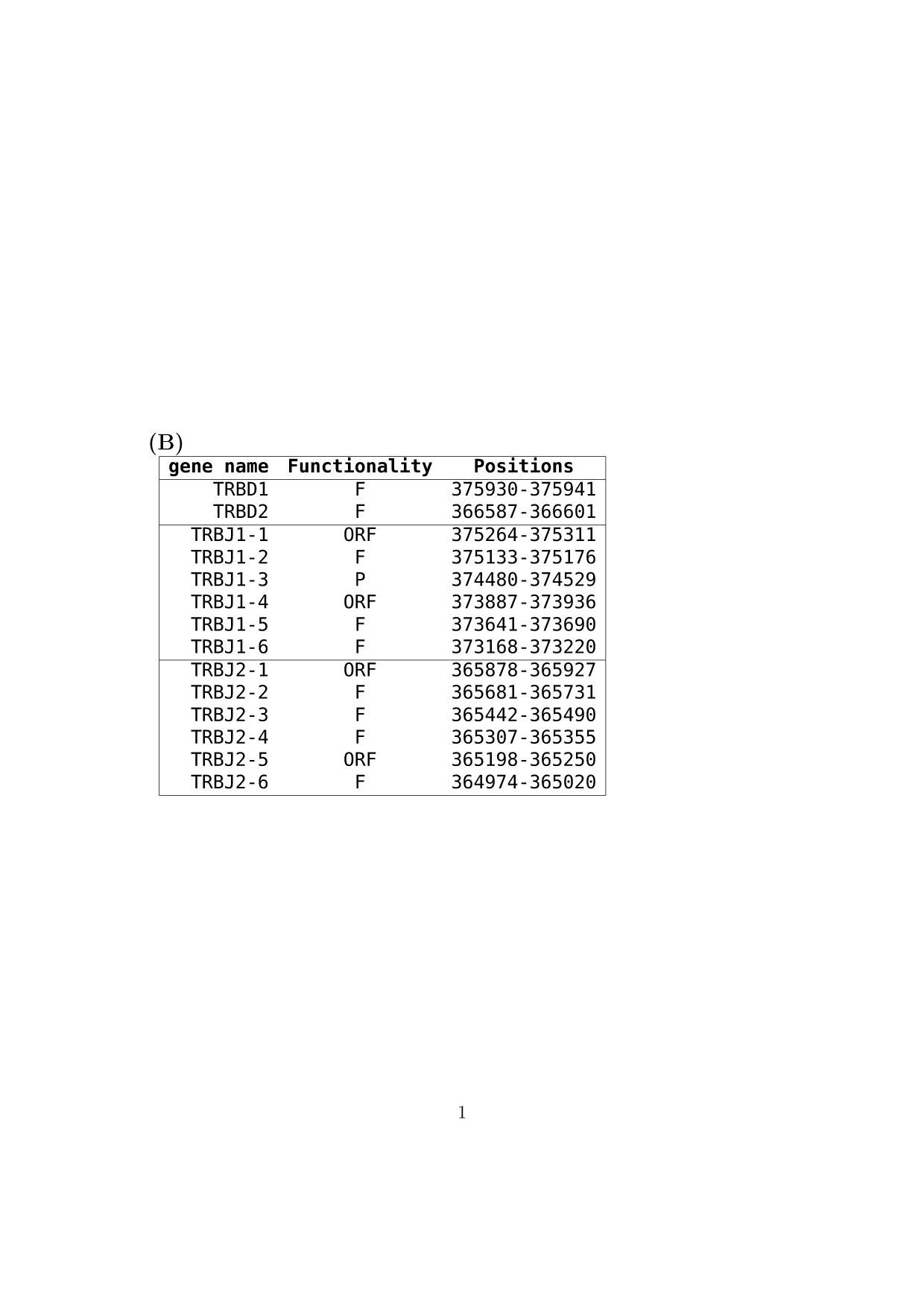

Supplement: Supplementary file 13 — (B) [file 251_2019_1142_Fig13_ESM.png]

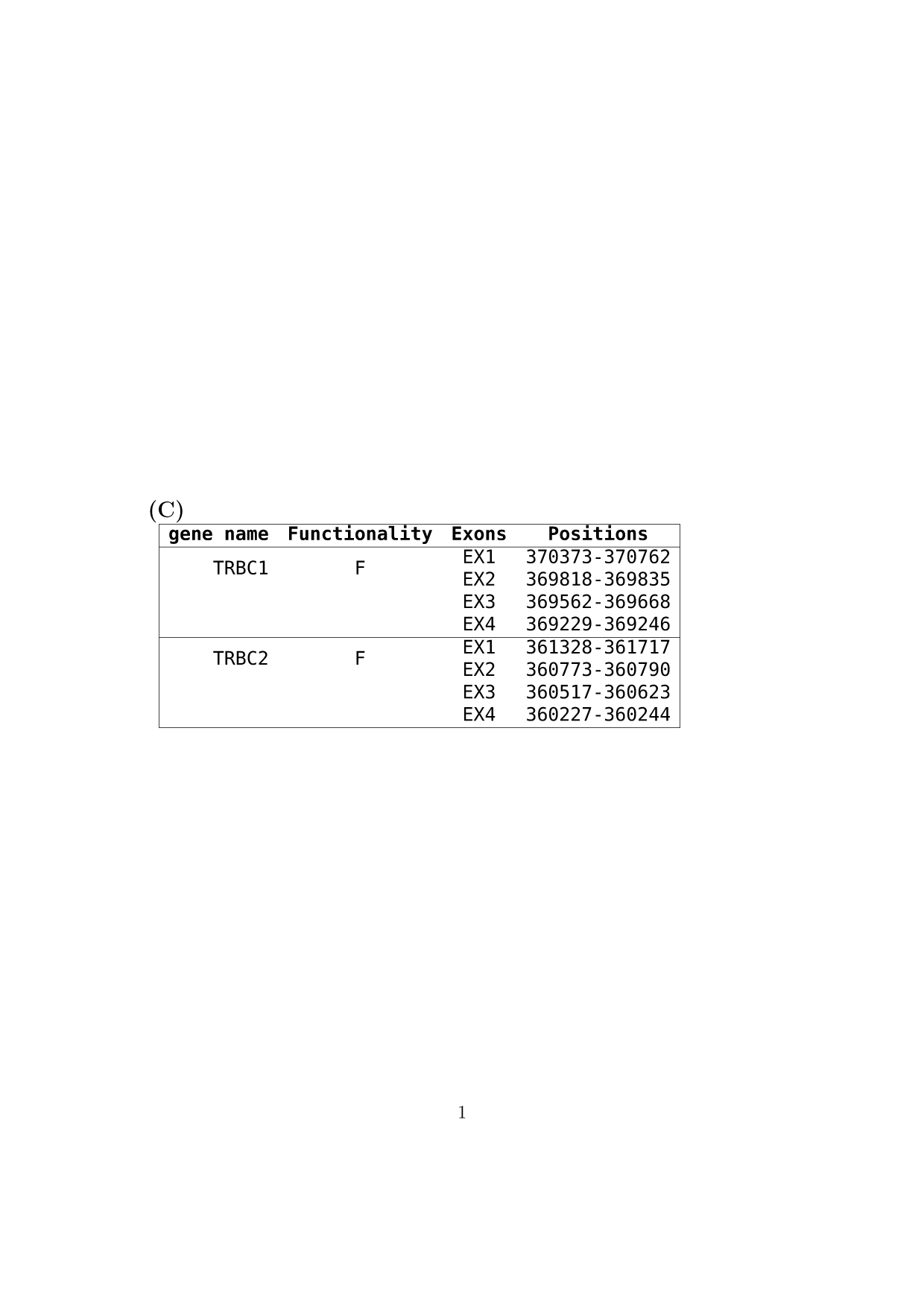

Supplement: Supplementary file 15 — (C) [file 251_2019_1142_Fig14_ESM.png]
